# Size of Pediatric Tracheostomy Tube and Predictors of Postoperative Complications

**DOI:** 10.1002/ohn.1306

**Published:** 2025-05-29

**Authors:** Soukaina Eljamri, Jordyn Lucas, Amber Shaffer, Basil Hashimi, Marina Rushchak, Reema Padia

**Affiliations:** ^1^ University of Pittsburgh School of Medicine Pittsburgh Pennsylvania USA; ^2^ Department of Otolaryngology–Head and Neck Surgery Macon and Joan Brock Virginia Health Sciences at Old Dominion University, Children's Hospital of the King's Daughters Norfolk Virginia USA; ^3^ UPMC Children's Hospital of Pittsburgh Pittsburgh Pennsylvania USA; ^4^ Department of Otolaryngology University of Utah Salt Lake City Utah USA

**Keywords:** decannulation, trachea, tracheostomy, tracheostomy tube, weight

## Abstract

**Objective:**

(1) To investigate weight as an alternative guide for trach tube choice. (2) Evaluate complications 3‐month posttracheostomy and associations with patient and trach tube factors.

**Study Design:**

Retrospective cohort study.

**Setting:**

Single academic medical center.

**Methods:**

Patients <2 years old who underwent tracheostomy placement from 2017 to 2022 were identified. Associations between trach characteristics, trach size/length, chest x‐ray (CXR) and intraoperative endoscopy measurements, and complications within 3 months posttracheostomy placement were evaluated.

**Results:**

In total, 68 patients were included in the study, with a median age of 4 months (range: 1 day to 1.74 years) and weight of 4.1 kg (range: 1.8‐11.6 kg) at the time of the procedure. The length of the trachea measured on CXR was more closely associated with weight (*ρ* = 0.403, *P* = .0007) than with age (*ρ* = 0.291, *P* = .02). Major complications, including accidental decannulation or death, occurred in 16/68 (24%) patients and minor events, including skin breakdown or difficult trach change, occurred in 19/68 (27.9%) patients and were not associated with age or weight. Major events were associated with male sex (*P* = .006) and shorter distance from the trach to the carina (*P* = .03).

**Conclusion:**

In pediatric patients with a tracheostomy, the anatomical distance between the thoracic inlet and carina on CXR was more strongly associated with weight rather than age. Postoperative complications were not associated with age or weight, but rather with male sex and shorter distance from the trach to the carina. When selecting a trach size for patients younger than 2 years, a weight‐based algorithm may aid in reducing posttracheostomy complications.

Pediatric tracheostomy placement can have potentially life‐threatening complications, including accidental decannulation, granulation tissue formation, ulceration, catastrophic bleeding, and tracheal stenosis. Selecting an appropriately sized tracheostomy tube is an important first step in preventing many of these adverse events. Inappropriately small tracheostomy tubes may lead to accidental decannulation or inadequate ventilation due to air leaking, whereas improperly large tubes may damage the tracheal wall causing ulceration, granulation tissue formation, and strictures.^1^ Inappropriately long tubes or those placed too close to the carina may induce bronchospasm or unintentional endobronchial intubation, leading to desaturations and subsequent respiratory compromise. Age‐based formulas have generally been adopted in clinical practice to predict tracheostomy tube size in pediatric patients, but alternative approaches have been suggested including weight‐based formulas, height‐based formulas, combination formulas, and custom pediatric tracheostomy tubes.^1^, ^2^, ^3^ Currently, the most commonly used approach for determining tracheostomy size is using patient age to estimate both inner tube diameter and tube length. To confirm appropriate tube placement, both intraoperative evaluation using flexible tracheoscopy and bedside confirmation using chest x‐ray (CXR) postoperatively are recommended.^1^, ^4^ This study investigated a weight‐based approach to tracheostomy tube sizing and predictors of postoperative tracheostomy complications in a cohort of pediatric patients 3 months after tracheostomy.

## Methods

This study was reviewed and approved by the University of Pittsburgh Institutional Review Board (STUDY19100247). We performed a retrospective review of pediatric patients who underwent a tracheostomy from January 2017 to January 2022 at a single tertiary pediatric hospital. Included patients were less than 2 years of age, underwent an open tracheostomy procedure by an otolaryngologist, and had at least 3 months of documented follow‐up. Patients who did not meet these criteria were excluded. Demographic data included biological sex, age in months, and weight in kilograms. Clinical characteristics included medical comorbidities, airway comorbidities, tracheostomy dressing details, and adverse events. Specific measurements were obtained for each patient including distance from the distal end of the tracheostomy tube to the carina in millimeters based on intraoperative measurements using flexible tracheoscopy and bedside measurements using CXR, trachea length in millimeters (distance from thoracic inlet to the carina) according to CXR, and tracheostomy tube size and length based on manufacturer description. Intraoperative tracheoscopy measurements were obtained from each patient's operative report. These measurements are obtained in the operating room (OR) by the otolaryngologist using the following approach: after placing the tracheostomy tube and applying standard tracheostomy dressings, the provider uses a flexible fiberoptic laryngoscope entered through the tube to identify the level of the carina. A finger is placed externally to keep note of this position before moving the scope back to the distal end of the tracheostomy tube. The distance between the finger placed on the carinal position and the point where the scope sits at the distal end of the tube is then measured in millimeters using a ruler and recorded in the operative report. CXR measurements were calculated manually by the study team members (divided between three individual members), using the clavicle to represent the thoracic inlet and using the measurement tool on ClinicView imaging software to obtain measurements from the clavicle to the carina and from the distal end of the trach tube to the carina. All team members were trained by a senior team member on how to properly collect CXR measurements, and all measurements were obtained using this standardized approach. Difficult to read CXRs were looked at by two different team members. For the trachea length measurement, the most recent preoperative CXR was used. For the distance from the distal end of the tracheostomy tube to the carina, the postoperative CXR was used. When obtaining the above measurements, it was not possible to account for differences in patient positioning, including neck extension, as this was not regularly documented in the operative report. Major complications were defined as any of the following: accidental decannulation, cardiac arrest, or death. Minor events were defined as stomal wound concerns, neck skin breakdown, difficult trach change, or trouble with ventilation. Major events are rare and often life‐threatening. Minor events are more common and seen at various stages of peristomal wound healing.^4^

Data were summarized as n (%) for categorical variables. Median (range) and mean (standard deviation, SD) were reported for nonnormally (Shapiro‐Wilk *P* < .05) and normally distributed continuous variables, respectively. Demographics and clinical characteristics associated with complications were evaluated using chi‐square, Fisher's exact, Wilcoxon rank‐sum, and *t* tests. Associations between tracheostomy measurements were evaluated using Spearman rank correlation. Binomial (“exact”) 95% confidence intervals (CIs) were calculated for categorical characteristics when comparing patients with and without complications. Multivariable logistic regression was conducted with major complications as the response variable and factors that were significantly associated on univariate analysis (sex, distance from the distal end of the trach tube to the carina on CXR, and tube diameter) as predictors. Analyses were performed using Stata/SE 16.0 (StataCorp) with *α* = .05.

## Results

### Patient Characteristics

A total of 68 patients were included in the study; they were nearly evenly distributed across biological sex (35/68, 52% male; 33/68, 49% female) with a median age of 4 months (range: 12 days to 1.60 years) and weight of 4.1 kg (range: 1.8‐9.0 kg) at the time of the procedure. Most patients had cardiac (39/68, 57%) or pulmonary (29/68, 43%) comorbidities, a preoperative diagnosis of ventilatory or respiratory difficulty (52/68, 77%), and OR diagnosis of subglottic stenosis (16/68, 24%) (Table 1). Most patients were sedated without the use of paralytics between the tracheostomy surgery and the first trach change (37/60, 62%). The median tracheostomy tube size was a 3.5 tracheostomy, and the median length of the tube was 34 mm. Mepilex was the most commonly used dressing type under the trach tube (43/58, 74.1%), followed by Hydrafera Blue (11/58, 19%) and Tritec (4/58, 6.9%). Duoderm was the most commonly used dressing around the trach tie skin site (45/60, 75%), with Mepilex (14/60, 23.3%) following and Coloplast used in one patient. Maturation of the stoma was documented in 6/68 (8.8%) of patients (Table 2).

**Table 1:** Demographics

Age, n (%)	
<2.5 mo	17/68 (25%)
2.5‐4.4 mo	17/68 (25%)
4.5‐7.3 mo	17/68 (25%)
7.4‐23 mo	17/68 (25%)
Biological sex, n (%)	
Male	35/68 (51.5)
Female	33/68 (48.5)
Age at time of procedure, y, median (range)	0.374 (0.0356‐1.60)
Weight at time of procedure, kg, median (range)	4.1 (1.8‐9.0)
Comorbidities, n (%)	
Bronchopulmonary dysplasia	13/68 (19.1)
Tracheomalacia	10/68 (14.7)
Cardiac	39/68 (57.4)
Gastrointestinal	11/68 (16.2)
Craniofacial	17/68 (25)
Pulmonary	29/68 (42.7)
Failure to thrive	3/68 (4.4)
Obstructive sleep apnea (OSA)	5/68 (7.4)
Genetic	15/68 (22.1)
Neurologic	5/68 (7.4)
Tracheoesophageal fistula	2/68 (2.9)
Preoperative diagnosis, n (%)	
Ventilatory/respiratory difficulty	52/68 (76.5)
Airway obstruction/severe OSA	26/68 (38.2)
Operating room diagnosis, n (%)	
Tracheomalacia	8/68 (11.8)
Bronchomalacia	6/68 (8.8)
Subglottic stenosis	16/68 (23.5)
Airway obstruction	6/68 (8.8)
Laryngomalacia	6/68 (8.8)
Vascular	2/68 (2.9)
Other	18/68 (26.5)
Vocal fold immobility	3/68 (4.4)
Vocal fold granuloma	6/68 (8.8)

**Table 2:** Trach Characteristics

Size of trach tube placed, median (range)	3.5 (3‐3.5)
Length of trach tube placed, mm, median (range)	34 (26‐44)
Trach length: CXR, mm, mean (SD)	29.0 (6.6)
Distance from trach to carina: CXR, mm, mean (SD)	18.2 (5.5)
Distance from trach to carina: OR, mm, mean (SD)	12.6 (5.5)
Type of trach, n (%)	
Bivona	68/68 (100)
Cuffed	10/68 (14.7)
Dressings under tracheostomy tube, n (%)	
Mepilex	43/58 (74.1)
Hydrafera Blue	11/58 (19)
Tritec	4/58 (6.9)
Stomal dressing, n (%)	
Duoderm	45/60 (75)
Mepilex	14/60 (23.3)
Coloplast	1/60 (1.7)
Stomal maturation, n (%)	6/68 (8.8)
Posttracheostomy sedation, n (%)	
Sedation only	37/60 (61.7)
Paralysis	23/60 (38.3)

Abbreviations: CXR, chest x‐ray; OR, operating room.

### Trach Measurements

**Figure 1:**
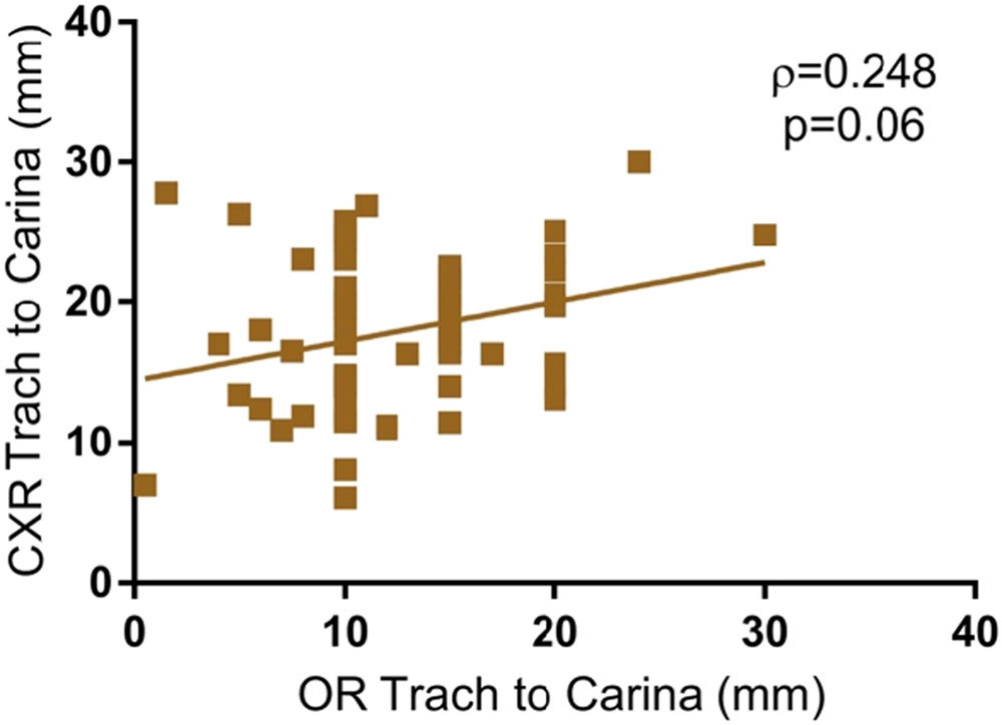
Distance from trach tube tip to the carina on chest x‐ray (CXR) versus tracheoscopy. Scatter plot of CXR and tracheoscopy measurements of distance between distal trach tube and carina, demonstrating no significant correlation between the two measurements (*P* = .06). OR, operating room.

**Figure 2:**
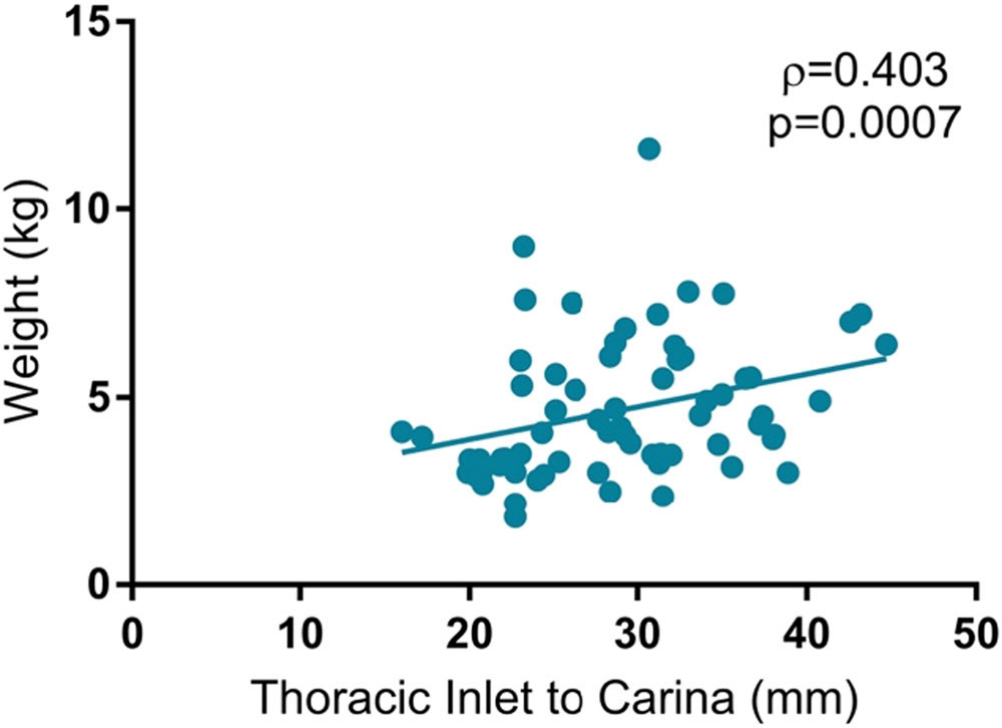
Patient weight and distance from the thoracic inlet to the carina. Scatter plot of patient weight and distance from the thoracic inlet to the carina, demonstrating a significant correlation between weight and distance (*P* = .0007).

**Figure 3:**
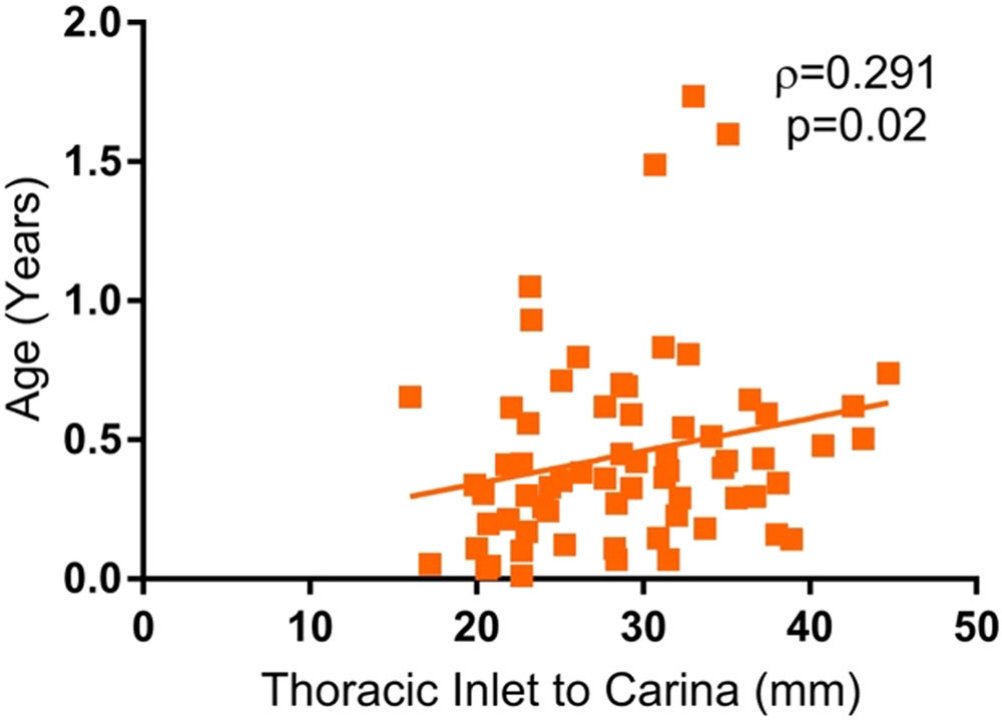
Patient age and distance from the thoracic inlet to the carina. Scatter plot of patient age and distance from the thoracic inlet to the carina, demonstrating a significant correlation between age and distance (*P* = .02).

The patient cohort had a mean distance of 12.6 mm (SD 5.5) from the distal end of the trach tube to the carina based on OR flexible tracheoscopy, 18.2 mm (SD 5.5) from the distal end of the trach tube to the carina based on postoperative CXR, and a mean tracheal length of 29.0 mm (SD 6.6) according to preoperative CXR (Table 2). The relationship between the distance from the distal end of the tracheostomy tube to the carina measured using flexible tracheoscopy and on CXR was not significant (*ρ* = 0.248, *P* = .06) (Figure 1). The length of the trachea measured on CXR was more closely associated with weight (*ρ* = 0.403, *P* = .0007) (Figure 2) than with age (*ρ* = 0.291, *P* = .02) (Figure 3).

### Major and Minor Complications

**Figure 4:**
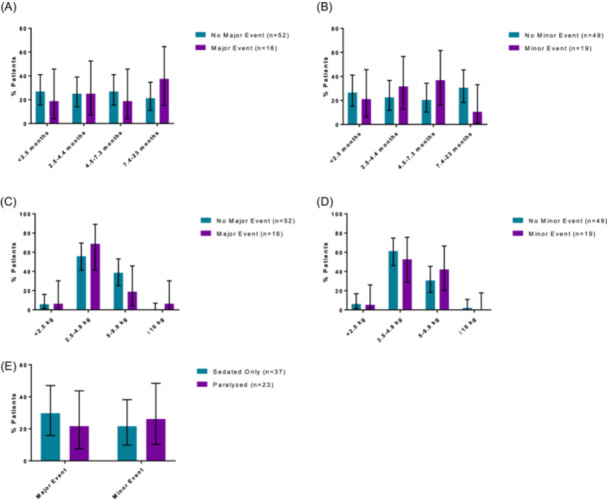
(A‐E) Prevalence of complications by patient age, weight, and sedation status. Bar graphs of prevalence of major (A) and minor (B) events by patient age across four age groups (<2.5 months, 2.5‐4.4 months, 4.5‐7.3 months, and 7.4‐23 months). Bar graphs of prevalence of major (C) and minor (D) events by patient weight across four weight groups (<2.5 kg, 2.5‐4.9 kg, 5‐9.9 kg, and >10 kg). Bar graphs of prevalence of major and minor events by sedation status: sedated only patients and paralyzed patients (E). Error bars indicate 95% confidence intervals.

**Figure 5:**
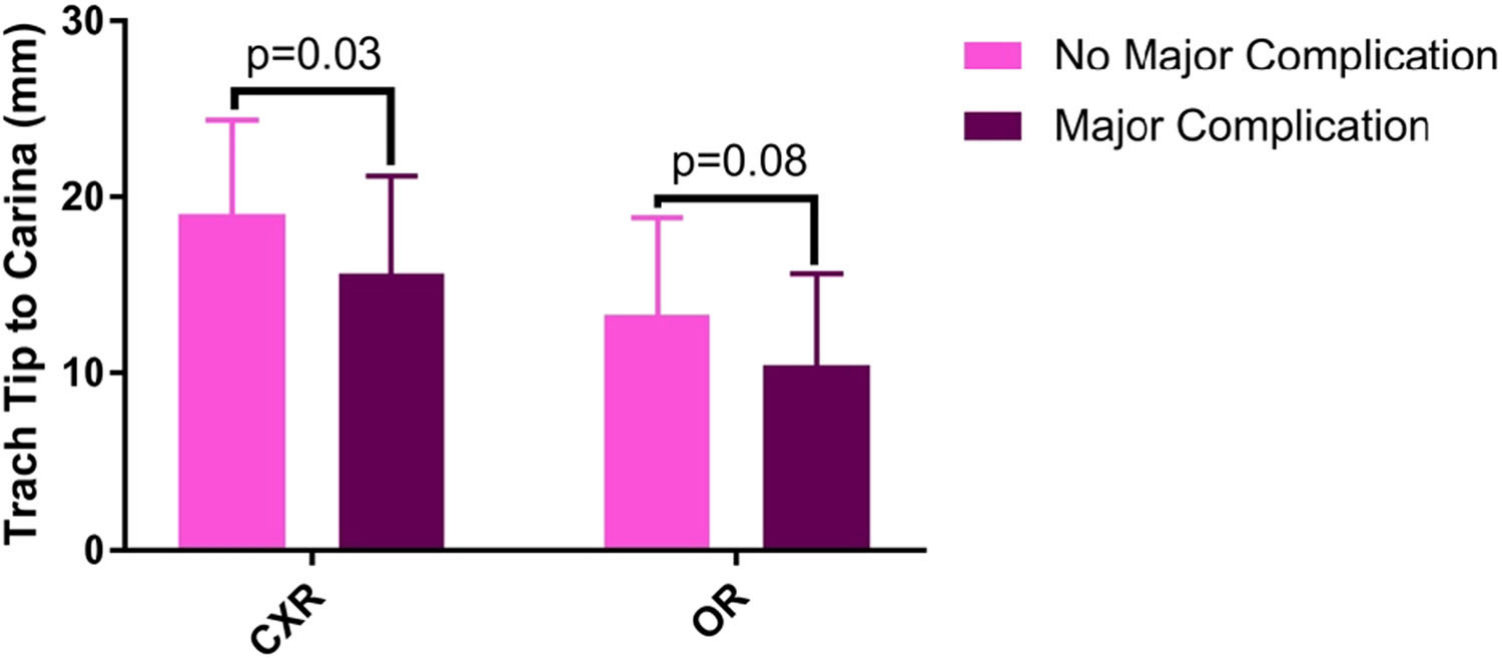
Postoperative complications and distance from trach tube tip to the carina. Mean distances from the distal end of the tracheostomy tube to the carina on chest x‐ray (CXR) or on tracheoscopy (operating room, OR) in those with and without complications. Error bars indicate standard deviations. Distance from the distal end of the tracheostomy tube to the carina on CXR was significantly shorter in those with major complications than those without major complications (*P* = .03).

Major complications, including accidental decannulation or death, occurred in 16/68 (24%) patients, and minor events, including skin breakdown or difficult trach change, occurred in 19/68 (27.9%) patients (Table 3). Events were analyzed in terms of patient weight, age, and sedation status (**Figures 4A‐E**). When analyzed as a continuous variable, patient age at procedure was not significantly associated with major (*P* = .7) or minor (*P* = .2) postoperative complications. Weight at procedure was also not associated with postoperative complications (major: *P* = .2; minor: *P* = .9) (Tables 4 and 5). Further, when the analysis was conducted with patients in subgroups based on age and weight, the relationship between these subgroups and postoperative complications remained nonsignificant. Major complications were observed in 13/35 (37%) males compared with 3/33 (9%) females (*P* = .006). Distance from the distal end of the tracheostomy tube to the carina on CXR was shorter in those with major complications (mean 15.7 mm, SD 5.5 mm) than those without major complications (mean 19.0 mm, SD 5.3 mm) (*P* = .03) (Figure 5). Distance from the distal end of the tracheostomy tube to the carina based on intraoperative tracheoscopy measurement was not associated with postoperative complications. Tracheostomy tube inner diameter was larger in those with major complications (median 3.5 mm, range 3‐4 mm) compared with those without major complications (median 3.5 mm, range 3‐3.5 mm) (*P* = .04) (Table 4). When a multivariable regression model was used to control for biological sex, the odds of major complications decreased as the distance from the distal end of the tracheostomy tube to the carina on CXR increased (odds ratio [OR]: 0.842, 95% CI: 0.731, 0.969) (Table 6). However, the association between tube diameter and major events was no longer significant. Posttracheostomy complications were not associated with stoma dressing type, skin dressing type, maturation of the stoma, or the use of postoperative paralytics (Tables 4 and 5).

**Table 3:** Complications

Major event, n (%)	
Decannulation	2/68 (2.9)
Code	3/68 (4.4)
Death	12/68 (17.7)
Minor event, n (%)	
Stoma erosion	10/68 (14.7)
Neck skin breakdown	4/68 (5.9)
Difficult trach change	4/68 (5.9)
Trouble with ventilation	3/68 (4.4)

**Table 4:** Univariate Associations With Any Major Event^a^

	No major event (N = 52)		Major event (N = 16)		
	n (%)	95% CI	n	95% CI	*P*
Age					.7
<2.5 mo	14/52 (26.9)	15.6‐41.0	3/16 (18.8)	4.1‐45.7	.7
2.5‐4.4 mo	13/52 (25.0)	14.0‐39.0	4/16 (25.0)	7.3‐52.4	1.0
4.5‐7.3 mo	14/52 (26.9)	15.6‐41.0	3/16 (18.8)	4.1‐45.7	.7
7.4‐23 mo	11/52 (21.2)	11.1‐34.7	6/16 (37.5)	15.2‐64.6	.2
Male	22/52 (42.3)	28.7‐56.8	13/16 (81.3)	54.4‐96.0	**.006**
Weight					.2
<2.5 kg	3/52 (5.8)	1.2‐16.0	1/16 (6.3)	0.2‐30.2	1
2.5‐4.9 kg	29/52 (55.8)	41.3‐69.5	11/16 (68.8)	41.3‐89.0	.4
5‐9.9 kg	20/52 (38.5)	25.3‐53.0	3/16 (18.8)	4.1‐45.7	.1
>10 kg	0/52 (0.0)	0.0‐6.9	1/16 (6.3)	0.2‐30.2	.2
Comorbidities					
Bronchopulmonary dysplasia	10/52 (19.2)	9.6‐32.5	3/16 (18.8)	4.1‐45.7	1
Tracheomalacia	8/52 (15.4)	6.9‐28.1	2/16 (12.5)	1.6‐38.4	1
Cardiac	28/52 (53.9)	39.5‐67.8	11/16 (68.8)	41.3‐89.0	.3
Gastrointestinal	7/52 (13.5)	5.6‐25.8	4/16 (25.0)	7.3‐52.4	.3
Craniofacial	13/52 (25.0)	14.0‐39.0	4/16 (25.0)	7.3‐52.4	1.0
Pulmonary	23/52 (44.2)	30.5‐58.7	6/16 (37.5)	15.2‐64.6	.6
Failure to thrive	1/52 (1.9)	0.01‐10.3	2/16 (12.5)	1.6‐38.4	.1
Obstructive sleep apnea (OSA)	5/52 (9.6)	3.2‐21.0	0/16 (0.0)	0.0‐20.6	.3
Genetic	11/52 (21.2)	11.1‐34.7	4/16 (25.0)	7.3‐52.4	.7
Neurologic	4/52 (7.7)	2.1‐18.5	1/16 (6.3)	0.2‐30.2	1.0
Tracheoesophageal fistula	1/52 (1.9)	0.01‐10.3	1/16 (6.3)	0.2‐30.2	.4
Pre‐op diagnosis					
Ventilatory/respiratory difficulty	39/52 (75.0)	61.1‐86.0	13/16 (81.3)	54.4‐96.0	.7
Airway obstruction/severe OSA	21/52 (40.4)	27.0‐54.9	5/16 (31.3)	11.0‐58.7	.5
Type of trach					
Cuffed	9/52 (17.3)	8.2‐30.3	1/16 (6.3)	0.2‐30.2	.4
Dressing under trach					
Mepilex	33/44 (75.0)	59.7‐86.8	10/14 (71.4)	41.9‐91.6	1.0
Hydrafera Blue	7/44 (15.9)	6.6‐30.1	4/14 (28.6)	8.4‐58.1	.4
Tritec	4/44 (9.1)	2.5‐21.7	0/14 (0.0)	0.0‐23.2	.6
Dressing around skin					
Duoderm	34/45 (75.6)	60.5‐87.1	11/15 (73.3)	44.9‐92.2	1.0
Mepilex	10/45 (22.2)	11.2‐37.1	4/15 (26.7)	7.8‐55.1	.7
Coloplast	1/45 (2.2)	0.1‐11.8	0/15 (0.0)	0.0‐21.8	1.0
Maturation of stoma	4/52 (7.7)	2.1‐18.5	2/16 (12.5)	1.6‐38.4	.6
OR findings					
Tracheomalacia	6/52 (11.5)	4.4‐23.4	2/16 (12.5)	1.6‐38.4	1.0
Bronchomalacia	5/52 (9.6)	3.2‐21.0	1/16 (6.3)	0.2‐30.2	1.0
Subglottic stenosis	15/52 (28.9)	17.1‐43.1	1/16 (6.3)	0.2‐30.2	.09
Airway obstruction	5/52 (9.6)	3.2‐21.0	1/16 (6.3)	0.2‐30.2	1.0
Laryngomalacia	5/52 (9.6)	3.2‐21.0	1/16 (6.3)	0.2‐30.2	1.0
Vascular	2/52 (3.9)	0.5‐13.2	0/16 (0.0)	0.0‐20.6	1.0
Other	16/52 (30.8)	18.7‐45.1	2/16 (12.5)	1.6‐38.4	.2
Vocal fold immobility	3/52 (5.8)	1.2‐16.0	0/16 (0.0)	0.0‐20.6	1.0
Vocal fold granuloma	5/52 (9.6)	3.2‐21.0	1/16 (6.3)	0.2‐30.2	1.0
Sedation after trach					
Paralyzed	18/44 (40.9)	26.3‐56.8	5/16 (31.3)	11.0‐58.7	.5

	Mean	SD	Mean	SD	*P*
Distance from trach to carina: OR	13.4	5.5	10.4	5.2	.08
Trach length: CXR	29.2	6.8	28.3	6.1	.6
Distance from trach to carina: CXR	19	5.3	15.7	5.5	**.03**

	Median	Range	Median	Range	*P*
Age at time of procedure, y	0.357	0.040‐1.60	0.427	0.003‐1.74	.3
Gestational age at birth, wk	34	23‐41	36.4	25‐40	.7
Weight at time of procedure, kg	4.3	1.8‐9.0	4	2.2‐11.6	.6
Ratio trach tube length to trach length CXR	1.2	0.8‐2.3	1.2	0.9‐2.3	.7
Size of trach tube placed	3.5	3‐3.5	3.5	3‐4	**.04**
Length of trach tube placed, mm	34	26‐44	34	28‐41	.4

Abbreviations: CXR, chest x‐ray; OR, operating room.

^a^ Bold value indicates *P* < .05.

**Table 5:** Univariate Associations With Any Minor Event

	No minor event (N = 49)		Minor event (N = 19)		
	n (%)	95% CI	n (%)	95% CI	*P*
Age					.2
<2.5 mo	13/49 (26.5)	15.0‐41.1	4/19 (21.1)	6.1‐45.6	.7
2.5‐4.4 mo	11/49 (22.5)	11.8‐36.6	6/19 (31.6)	12.6‐56.6	.5
4.5‐7.3 mo	10/49 (20.4)	10.2‐34.3	7/19 (36.8)	16.3‐61.6	.2
7.4‐23 mo	15/49 (30.6)	18.3‐45.4	2/19 (10.5)	1.3‐33.1	.1
Male	25/49 (51.0)	36.3‐65.6	10/19 (52.6)	28.9‐75.6	.9
Weight					.9
<2.5 kg	3/49 (6.1)	1.3‐16.9	1/19 (5.3)	0.1‐26.0	1.0
2.5‐4.9 kg	30/49 (61.2)	46.2‐74.8	10/19 (52.6)	28.9‐75.6	.5
5‐9.9 kg	15/49 (30.6)	18.3‐45.4	8/19 (42.1)	20.3‐66.5	.4
>10 kg	1/49 (2.0)	0.1‐10.9	0/19 (0.0)	0.0‐17.7	1.0
Comorbidities					
Bronchopulmonary dysplasia	9/49 (18.4)	8.8‐32.0	4/19 (21.1)	6.1‐45.6	1.0
Tracheomalacia	7/49 (14.3)	5.9‐27.2	3/19 (15.8)	3.4‐39.6	1.0
Cardiac	29/49 (59.2)	44.2‐73.0	10/19 (52.6)	28.9‐75.6	.6
Gastrointestinal	5/49 (10.2)	3.4‐22.2	6/19 (31.6)	12.6‐56.6	.06
Craniofacial	14/49 (28.6)	16.6‐43.3	3/19 (15.8)	3.4‐4.0	.4
Pulmonary	19/49 (38.8)	25.2‐53.8	10/19 (52.6)	28.9‐75.6	.3
Failure to thrive	2/49 (4.1)	0.1‐14.0	1/19 (5.3)	0.1‐26.0	1.0
Obstructive sleep apnea (OSA)	5/49 (10.2)	3.4‐22.2	0/19 (0.0)	0.0‐17.7	.3
Genetic	13/49 (26.5)	15.0‐41.1	2/19 (10.5)	1.3‐33.1	.2
Neurologic	2/49 (4.1)	0.5‐14.0	3/19 (15.8)	3.4‐39.6	.1
Tracheoesophageal fistula	1/49 (2.0)	0.1‐10.9	1/19 (5.3)	0.1‐26.0	.5
Pre‐op diagnosis					
Ventilatory/respiratory difficulty	37/49 (75.5)	61.1‐86.7	15/19 (79.0)	54.4‐94.0	1.0
Airway obstruction/severe OSA	20/49 (40.8)	27.0‐55.8	6/19 (31.6)	12.6‐56.6	.5
Type of trach					
Cuffed	6/49 (12.2)	4.6‐24.8	4/19 (21.1)	6.1‐45.6	.4
Dressing under trach					
Mepilex	29/43 (67.4)	51.5‐80.9	14/15 (93.3)	68.1‐99.8	.08
Hydrafera Blue	11/43 (25.6)	13.5‐41.2	0/15 (0.0)	0.0‐21.8	.05
Tritec	3/43 (7.0)	1.5‐19.1	1/15 (6.7)	0.2‐32.0	1.0
Dressing around skin					
Duoderm	32/44 (72.7)	57.2‐85.0	13/16 (81.3)	54.4‐96.0	.7
Mepilex	12/44 (27.3)	15.0‐42.8	2/16 (12.5)	1.6‐38.4	.3
Coloplast	0/44 (0.0)	0.0	1/16 (6.3)	0.2‐30.2	.3
Maturation of stoma	3/49 (6.1)	1.3‐16.9	3/19 (15.8)	3.4‐39.6	.3
OR findings					
Tracheomalacia	5/49 (10.2)	3.4‐22.2	3/19 (15.8)	3.4‐39.6	.7
Bronchomalacia	5/49 (10.2)	3.4‐22.2	1/19 (5.3)	0.1‐26.0	1.0
Subglottic stenosis	8/49 (16.3)	7.3‐29.7	8/19 (42.1)	20.3‐66.5	.05
Airway obstruction	5/49 (10.2)	3.4‐22.2	1/19 (5.3)	0.1‐26.0	1.0
Laryngomalacia	5/49 (10.2)	3.4‐22.2	1/19 (5.3)	0.1‐26.0	1.0
Vascular	1/49 (2.0)	0.1‐10.9	1/19 (5.3)	0.1‐26.0	.5
Other	15/49 (30.6)	18.3‐45.4	3/19 (15.8)	3.4‐39.6	.4
Vocal fold immobility	2/49 (4.1)	0.5‐14.0	1/19 (5.3)	0.1‐26.0	1.0
Vocal fold granuloma	4/49 (8.2)	2.3‐19.6	2/19 (10.5)	1.3‐33.1	1.0
Sedation after trach					
Paralyzed	17/46 (37.0)	23.2‐52.5	6/14 (42.9)	17.7‐71.1	.8
Major events					.5
Decannulation	2/49 (4.1)	0.5‐14.0	0/19 (0.0)	0.0‐17.7	1.0
Code	2/49 (4.1)	0.5‐14.0	1/19 (5.3)	0.1‐26.0	1.0
Death	10/49 (20.4)	10.2‐34.3	2/19 (10.5)	1.3‐33.1	.5

	Mean	SD	Mean	SD	*P*
Distance from trach to carina: OR	13.3	5.5	10.9	5.3	.1
Trach length: CXR	28.8	7.2	29.4	5.1	.7
Distance from trach to carina: CXR	17.6	5.7	19.9	4.7	.1

	Median	Range	Median	Range	*P*
Age at time of procedure, y	0.400	0.003‐1.74	0.353	0.068‐0.712	.7
Gestational age at birth, wk	36	23‐41	32	23‐40	.08
Weight at time of procedure, kg	4	1.8‐11.6	4.7	2.4‐6.8	.5
Ratio trach tube length to trach length CXR	1.2	0.8‐2.3	1.1	0.8‐1.8	.3
Size of trach tube placed	3.5	3‐4	3.5	3‐3.5	.6
Length of trach tube placed, mm	34	28‐44	34	26‐44	.3

Abbreviations: CXR, chest x‐ray; OR, operating room.

**Table 6:** Multivariable Analysis of Factors Impacting Risk of Major Event^a^

	OR	95% CI	*P*
Size of trach tube placed	13.3	0.346, 508	**.2**
Male (sex)	7.69	1.64, 36.1	**.01**
Distance from trach to carina: CXR	0.842	0.731, 0.969	**.02**

Abbreviations: CI, confidence interval; CXR, chest x‐ray; OR, odds ratio.

^a^ Bold value indicates *P* < .05.

## Discussion

In this cohort of pediatric patients posttracheostomy, the length of the trachea, estimated by distance from the thoracic inlet to the carina on CXR, was significantly associated with both age and weight, but had a stronger correlation to weight. Age and weight were not associated with major or minor complications in this cohort, and the relationship remained insignificant when analyzing patients in subgroups based on age and weight. Ultimately, our study suggests that a weight‐based formula may improve efficacy in estimating appropriate sizing of tracheostomy tubes in pediatric patients, but neither weight nor age accounts for the incidence of postoperative complications.

Although age and weight were both significantly associated with the distance between the thoracic inlet and the carina on CXR, weight was much more significantly correlated. This finding offers potential insight into why weight‐based formulas or combination formulas that incorporate weight may provide a more reliable prediction of tracheostomy tube size. In other words, weight‐based formulas may capture the patient's underlying anatomy more accurately, especially in neonates with low‐weight, growth restriction, or other developmental abnormalities who may not necessarily align with the predetermined sizing predictions of their age group. This finding correlates with a 2019 retrospective study of 171 pediatric patients, where weight was found to be the only statistically significant predictor of cross‐sectional tracheal area and the most significant predictor of tracheal AP diameter when compared to airway area calculations from CT or MRI imaging.^5^ Our findings also align with those of another 2019 study, which demonstrated that an age‐based formula provided accurate tracheostomy tube size predictions in 58% of patients, but when weight was incorporated into the formula, the prediction accuracy increased to 65%.^6^ Together, these findings suggest that weight may be a more accurate predictor of underlying airway anatomy compared to age in pediatric patients.

In terms of major and minor complications, major events were associated with male sex and shorter distance from the trach to the carina. Minor events were not significantly associated with any variables. Age, weight, clinical comorbidities, sedation status, and tracheostomy dressings were not associated with any major or minor events. Despite the correlation with major events, being male was not significantly associated with any comorbidity within our cohort, meaning these patients were not initially sicker compared to female patients in our cohort. Thus, the mechanism for why being male is associated with more complications remains unclear. Our findings also demonstrated that a shorter distance from the end of the trach tube to the carina was associated with more major complications, including accidental decannulation, death, or code.

Although it may be hypothesized that a trach tube positioned closer to the carina would be less likely to be dislodged, an inappropriately long tracheostomy tube may sit closer to the carina, inducing bronchospasm and respiratory compromise. Finally, a larger trach tube diameter was also associated with major complications, but this finding was no longer significant when controlled for biological sex. Although a larger tube trach tube diameter would be expected to be less likely to dislodge, one potential explanation for this may be that a larger tube may be more difficult to replace once dislodged, thus increasing the risk of coding events and death.

There remains a gap in the literature regarding risk factors predicting tracheostomy complications. A 2023 study, which explored factors associated with accidental decannulation in pediatric patients, found that risk factors for accidental decannulation included the use of smaller tracheostomy tubes (<4 mm in diameter), lower staff supervision, and the ability of the patient to reach the midline with their hands from a seated position. It is important to note that in this study, the authors found that self‐decannulation was the primary mechanism for decannulation events, so the three risk factors identified may be mediating this particular mechanism.^7^ Identifying risk factors for tracheostomy complications is instrumental in optimizing surgical planning, timing, and approach, and postoperative management to prevent adverse events.

When evaluating appropriate tracheostomy placement, it is also important to consider both intraoperative bronchoscopy measurements and bedside CXR measurements to confirm proper tracheal placement, which is recommended to be at least 2 to 3 rings above the carina.^1^ In this study, major tracheostomy complications, including death and decannulation, were associated with shorter bedside CXR measurements of the distance from the distal end of the tracheostomy tube to the carina compared to those without these complications. This same relationship was not observed with the intraoperative tracheoscopy measurement of this distance, suggesting that CXR measurements of tube position may be more predictive of postoperative complications than intraoperative measurements. There was also no correlation between measurements of trach tube placement (distance from the distal end of the tracheostomy tube to the carina) using flexible tracheoscopy and CXR, with the distance being longer on average on CXR measurement. These differences may be explained by changes in tube position during patient transfers from the operating table. In standard practice in pediatric patients, the tracheostomy tube is no longer sutured to the skin after placement because the tube may appear in place even during accidental decannulation and thus go unnoticed, especially as the child would not be able to communicate this appropriately.^1^ Nevertheless, this increases the risk of tube repositioning during patient transfers or external manipulation. Additionally, in the OR, patient repositioning and the application of dressings underneath the tracheostomy tube after placement may also explain the discrepancy between the tracheoscopy and CXR measurements. This highlights the importance of maintaining a neutral patient position before confirming trach placement with tracheoscopy or CXR.

Multiple studies in the literature, on both adult and pediatric patients, have debated whether the CXR after tracheostomy is a necessary step in the management of these patients.^8^, ^9^, ^10^, ^11^, ^12^ These studies have suggested that in large cohorts of patients, the posttracheostomy CXR rarely identifies any complications and should be used only in high‐risk patients, rather than routinely in all patients. A 2001 retrospective study on 101 pediatric patients found that only 6 patients had postoperative complications identified on CXR including either pneumothorax, pneumomediastinum, or both, and only 3 patients required intervention for these findings.^12^ Similarly, another 2001 retrospective study on 200 pediatric patients found that only 5 patients had a postoperative CXR finding, 3 of which required intervention. The study determined that the postoperative CXR has clinically significant findings in high‐risk patients, which were defined as the following: age less than 2 years, weight less than 17 kg, patient undergoing emergent tracheostomy, or patients undergoing tracheostomy with concomitant central line placement.^8^ Currently, per recommendations from the American Academy of Otolaryngology–Head and Neck Surgery, postoperative CXR after tracheostomy remains optional. As demonstrated in this study, there was no correlation between measures of tracheostomy tube placement on flexible tracheoscopy and CXR. Further, major complications in our patients were significantly correlated with CXR measurements of tube position and not with intraoperative tracheoscopy measurements. Although postoperative CXR is associated with increased radiation exposure and healthcare costs, our study provides evidence that some patient groups may continue to benefit from postoperative CXR to confirm appropriate placement and rule out associated complications.

This study has important limitations. Given the retrospective nature of this study, other variables that may have contributed to postoperative complications were not controlled for in this patient cohort, including length of intensive care unit/hospital stay, body mass index, and neck circumference. Further, anatomic variations amongst patients may have contributed to accidental decannulation events or other complications that may have required customized tube placement. Finally, both bronchoscopy measurements and CXR measurements of tracheostomy tube position in relation to the carina are based on estimations that are susceptible to human error and difficult to adjudicate for interrater reliability. Given these limitations, it is important to consider a prospective evaluation of a weight‐based algorithm to limit confounders in future work.

Accurate tracheostomy tube sizing is an easily modifiable variable in limiting adverse events. Weight‐based formulas for tracheostomy tube sizing may provide a more reliable estimate of the underlying patient anatomy as CXR measurements of the tracheal length were more closely associated with weight than age in our study. Multiple studies have identified weight‐based formulas for determining tracheal tube size in pediatric patients.^3^, ^6^ Employing these formulas, especially in patients less than 2 years of age, where weight may be a better predictor of anatomy than age, may prevent adverse events related to tube size. Further, although tube placement is confirmed intraoperatively via tracheoscopy, our findings emphasize the importance of confirming placement using bedside CXRs postoperatively as the tube may become dislodged during patient transfers. Tracheostomy tubes are becoming more prevalent in the United States with an estimated 2% of pediatric patients undergoing this procedure with an overall decreasing rate of permanent decannulation. As more patients receive tracheostomy tubes and retain those tubes for a longer period of time, with an average estimated cannulation time of 2 years, it is important to incorporate a standardized approach to tracheostomy tube sizing to prevent fatal complications.^1^

## Conclusion

Numerous approaches have been suggested for appropriate tracheostomy tube sizing, including age and weight‐based formulas. In this study, the anatomical distance between the thoracic inlet and carina on CXR was more strongly associated with weight rather than age, suggesting that weight may be more predictive of underlying airway anatomy. Postoperative complications in our cohort were not associated with age or weight, but rather with male sex and shorter distance from the trach to the carina. In future studies, a prospective evaluation of a weight‐based algorithm for tracheostomy tube size selection and comparison of intraoperative tracheoscopy findings and CXR calculations may aid in reducing posttracheostomy complications.

## Author Contributions

**Soukaina Eljamri**, data curation, investigation, methodology, writing – original draft preparation, review and editing; **Jordyn Lucas**, conceptualization, data curation, formal analysis, investigation, methodology, writing – original draft preparation, review and editing; **Amber Shaffer**, data curation, formal analysis, methodology, writing – review and editing; **Basil Hashimi**, data curation, methodology, writing – review and editing; **Marina Rushchak**, data curation, methodology; **Reema Padia**, conceptualization, investigation, methodology, project administration, supervision, writing – review and editing.

## Disclosures

### Competing interests

The authors have no conflicts of interest to declare.

### Funding source

The authors have no financial disclosures.
